# Characterization of G-Quadruplexes in Enterovirus A71 Genome and Their Interaction with G-Quadruplex Ligands

**DOI:** 10.1128/spectrum.00460-22

**Published:** 2022-04-21

**Authors:** Lu Lv, Leiliang Zhang

**Affiliations:** a Department of Clinical Laboratory Medicine, The First Affiliated Hospital of Shandong First Medical University & Shandong Provincial Qianfoshan Hospital, Jinan, Shandong, China; b Department of Pathogen Biology, School of Basic Medical Sciences, Shandong First Medical University & Shandong Academy of Medical Sciences, Jinan, Shandong, China; c Medical Science and Technology Innovation Center, Shandong First Medical University & Shandong Academy of Medical Sciences, Jinan, Shandong, China; Wuhan Institute of Virology

**Keywords:** human enteroviruses, EV-A71, G-quadruplexes, PQS, BRACO-19

## Abstract

Human enteroviruses cause many diseases; however, there is no specific therapeutic drug. G-quadruplex is an atypical secondary structure formed in the guanine rich region of DNA or RNA, which can exist in the viral genome. The different positions of G-quadruplex play an important role in the regulation of virus replication and infection. Whether G-quadruplexes are present in human enteroviruses is unknown. In current study, we analyzed the potential quadruplex forming sequences of human enteroviruses, especially EV-A71 virus, which causes hand, foot, and mouth disease. The results showed that there were a certain number of potential quadruplex-forming sequences in human enteroviruses. Through a variety of experimental methods, we evaluated the formation potential of EV-A71 encoded G-quadruplex and analyzed the binding ability of G-quadruplex ligands, including BRACO-19, pyridostatin and TMPyP4 to virus encoded G-quadruplexes. G-quadruplex ligands BRACO-19, PDS and TMPyP4 could inhibit the transcription of constructs containing EV-A71 G-quadruplex sequences. Moreover, we found that BRACO-19 was able to inhibit the replication of EV-A71, suggesting that targeting G-quadruplexes in EV-A71 genome by G-quadruplex ligands could be a novel antiviral way against EV-A71. Our finding not only uncovered the G-quadruplexes in human enteroviruses, but also would provide a new strategy for human enteroviruses therapy.

**IMPORTANCE** G-quadruplex is a stable nucleic acid secondary structure formed by the folding of guanine rich nucleic acid. The important regulatory function of G-quadruplex makes it an attractive target of antiviral effect. Human enteroviruses cause a variety of human diseases, including common cold, nervous system diseases, cardiovascular damage, and diabetes. Enterovirus A71 (EV-A71) is one of pathogens causing hand, foot, and mouth disease; however, whether G-quadruplexes are present in the genomes of human enteroviruses is unknown. The function of G-quadruplexes in the EV-A71 genomes is not clear. We predicted and characterized G-quadruplex sequences in EV-A71. G-quadruplex ligands were identified to stabilize EV-A71 G-quadruplexes with high affinities. We also demonstrated G-quadruplex ligand BRACO-19 inhibited EV-A71 replication. Our studies provide a framework for targeting G-quadruplexes in the enteroviruses genome, which will be a new way to develop antiviral agents against human enteroviruses.

## INTRODUCTION

Human enteroviruses (HEVs) cause a variety of human diseases, from common cold to nervous system diseases (such as encephalitis, aseptic meningitis and relaxation paralysis) (1), cardiovascular damage (viral myocarditis and dilated cardiomyopathy) (2), and metabolic diseases (such as diabetes) (3). HEVs include more than 100 serotypes, belonging to four enteroviruses (EV-A to EV-D) and three rhinoviruses (Rhinovirus A, Rhinovirus B, and Rhinovirus C) (4). EV-A was mainly related to hand, foot, and mouth disease (HFMD), including 22 serotypes such as Coxsackie virus A6, Coxsackie virus A16 and enterovirus A71 (EV-A71) (5). EV-A71 genome is about 7.4 kb, containing an open reading frame (ORF). The genome encodes a 220 kDa polyprotein precursor, which is hydrolyzed into four structural proteins (VP1, VP2, VP3 and VP4) and seven nonstructural proteins (2A, 2B, 2C, 3A, 3B, 3C and 3D) (6). EV-B is the largest enterovirus species, composed of 63 viruses, including Coxsackie virus B1-B6, 7 ECHO viruses and 50 other serotypes (7). EV-C includes three polioviruses and 20 other serotypes, including EV-C105, which is related to recent cases of acute flaccid myelitis in children (8). EV-D contains EV-D68, EV-D70 and EV-D94 (9). In addition to the successful PV vaccination in the 1950s, there is no prevention or treatment for most enterovirus infections.

**FIG 1:**
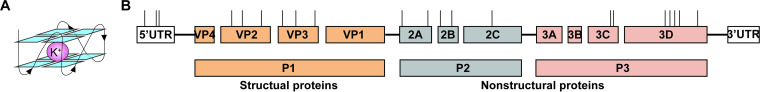
Schematic representation of the position of PQSs in the EV-A71 genome. (A) Schematic diagram of G-quadruplex structure. Guanines forming G-tetrad structure with a potassium ion inside through *Hoogsteen*-type hydrogen bonds. (B) EV-A71 genome diagram showing the sites of putative G-quadruplex sequences.

Viral genome plays a vital role in the life cycle of virus and provides an effective target for virus detection. A large number of studies have shown that some G-rich sequences in the virus genome can form a stable G-quadruplex (G4) structure and play a key role in the life cycle of the virus (10). G4 is a stable nucleic acid secondary structure formed by the folding of guanine rich nucleic acid (Fig. 1A) (11). Among them, four guanines form a G-quartet planar structure through *Hoogsteen* hydrogen bond, and more than two G-quartets form a stable G4 structure through π-π stacking. There are a large number of potential quadruplex-forming sequences (PQS) in the genome, so its biological function has attracted extensive attention. In recent years, with the discovery of G4 related binding proteins, the biological functions of G4 structure have been further explored (10). It is considered to participate in the regulation of a variety of biological functions and play a key role in important cell physiological processes. In addition, more and more attention has been paid to the characteristics and functionality of G4 structure. Nucleic acid nanostructures and biosensor methods based on G4 have also developed rapidly, and are widely used in targeting recognition, signal conversion and amplification (12).

Small molecule ligands could increase the stability of G-quadruplex or induce its formation. The binding modes of small molecule ligands with G-quadruplex are roughly divided into three types: (1) stacking on the surface of G-quadruplex; (2) inserting into the middle of the plane of the G-tetrad; (3) groove bound to G-quadruplex. Screening and designing small molecule ligands with high selectivity, low toxic and side effects and stable G-quadruplex as anti-cancer drug lead compounds has attracted attention in the field of cancer treatment. The reported compounds include acridine derivatives, pyridine derivatives, telomerstatin, imidazole derivatives, anthraquinone derivatives, berberine derivatives, porphyrin compounds, Indo quinoline derivatives, quinolone derivatives, quinoline derivatives, isandigotone derivatives, etc. At present, there are few targeted G-quadruplex drugs entering the clinical stage. Among them, guanine rich DNA aptamer and oligonucleotide molecule AS1411 are in the phase II clinical research stage of acute myeloid leukemia (13). CX-3543 (Quarfloxin) is in the phase II clinical research stage of neuroendocrine cancer (14).

The G4 structure in the viral genome is a potential viral inhibition target and biological imaging target. Molecular biological analysis shows that there is a highly conserved G4 structure in human immunodeficiency virus 1 (HIV-1) sequence (15). The G4 specific ligand can bind to the G4 structure in HIV-1 genome and inhibit the transcription and reversal of the virus (16). Genome-wide analysis in herpesvirus genome identified a large number of potential G4s (17). The G4 ligand BRACO-19 enhances the stability of G4 structure in Epstein-Barr virus (EBV) and inhibits the synthesis of EBV nuclear antigen 1 (EBNA1), a key protein in maintenance of the virus genome during latency (18). Studies have shown that there is a highly conserved positive parallel G4 structure in the RNA genome of hepatitis C virus (HCV) (19). G4 ligand specifically binds HCV G4 and effectively stabilizes its structure, thereby reducing HCV RNA replication and inhibiting the transcription of related proteins. In addition, there are potential G4 sequences in the promoter region of hepatitis B virus preS2/S gene (20). Through bioinformatics studies, biophysical methods, and molecular biological techniques, we identified many G4s in the genome of SARS-CoV-2 (21). G4s also exist in the genomes of other human pathogens, including human papillomavirus (22), Nipah virus (PMID: 32001794), Zika virus (23), dengue virus (24), influenza A virus (25), ebola virus (26), chikungunya virus (27), and human adenovirus (28). Since G4 structure exists in the genome of many viruses and participates in the key physiological processes of viruses, small molecule G4 ligands have become an important tool to explore the complex mechanism of virus life cycle.

The important regulatory function of G-quadruplex makes it an attractive target of antiviral effect. However, whether G-quadruplexes are present in the genomes of human enteroviruses is unknown. To address this question, we analyzed human enteroviruses RNA genomes and predicted the G-quadruplexes. Through experimental methods, we validated G-quadruplexes in EV-A71. This study will provide new ideas and methods for human enteroviruses treatment.

## RESULTS

### Identification and selection of PQSs conserved in the genomes of the Enterovirus family and EV-A71 strains.

**FIG 2:**
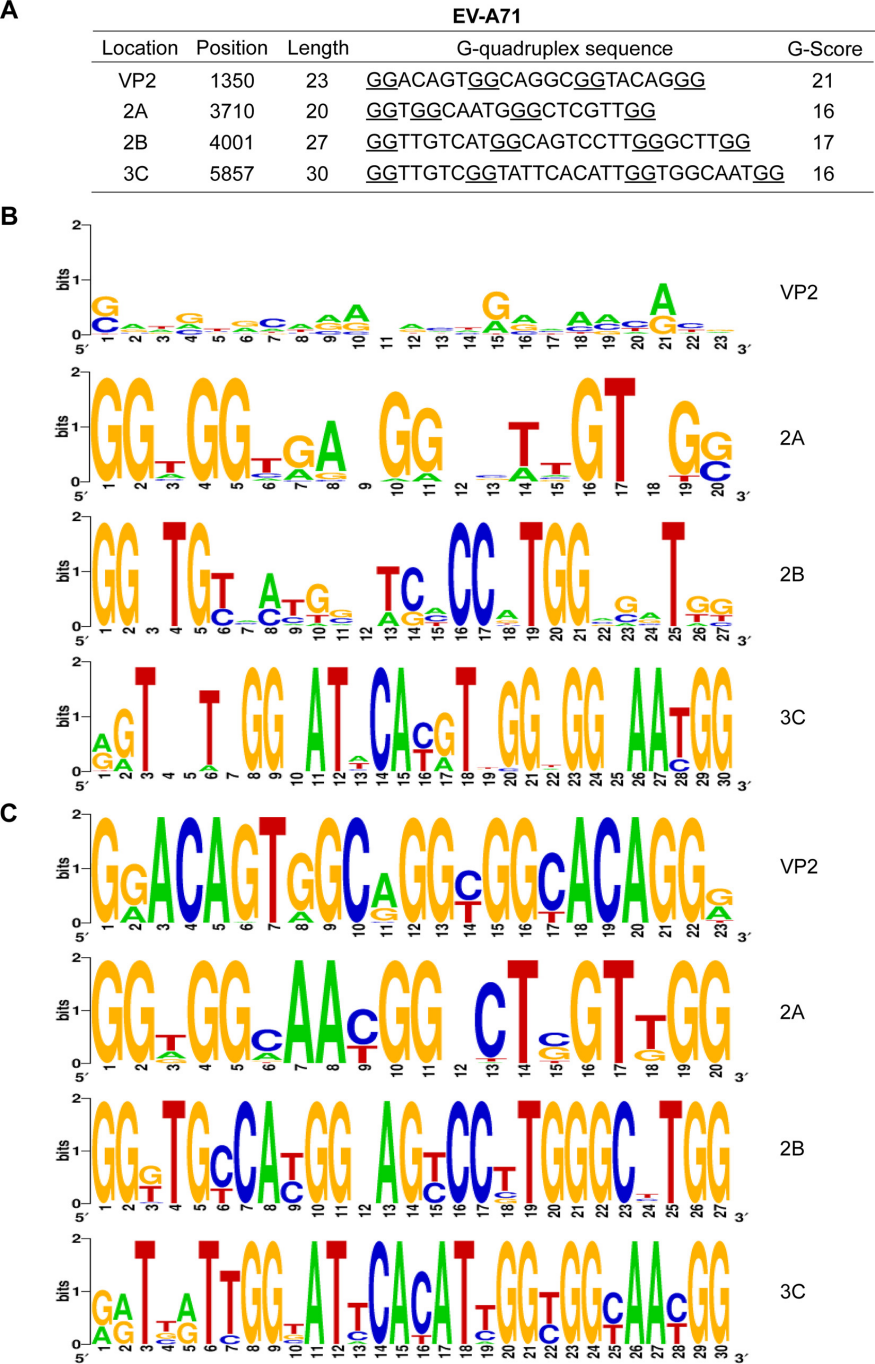
Conservation analysis of representative PQSs in the EV-A71 genome. (A) The selected G-quadruplex sequences at EV-A71 strain FY23 genome, in which the G-tracts are underlined. The four G-quadruplex sequences used in the study are distributed in the VP2, 2A, 2B, and 3C regions. (B-C) WebLogo images of the four G-quadruplex sequences showing that the conservation of particular nucleotides among the Enterovirus family (B) and different EV-A71 strains (C).

We first employed bioinformatics analysis to search for PQSs in the enterovirus genomes using QGRS mapper tool online (29). There was an aggregate of 21 PQSs in the EV-A71 strain FY23 genome, of which the coding region contained 18 G-quadruplex sequences and the noncoding region contained 3 G-quadruplex sequences (Fig. 1B). In accordance with G-score as well as the length of sequences, we focused on four representative sequences located in coding regions such as VP2, 2A, 2B, and 3C (Fig. 2A). EV-A71 belongs to the Enterovirus family, which has been divided into seven species: Enterovirus A, Enterovirus B, Enterovirus C, Enterovirus D, Rhinovirus A, Rhinovirus B, and Rhinovirus C (4). Numerous strains of EV-A71 have been discovered. We further explored PQSs conversation in the genomes of the Enterovirus family and EV-A71 strains. As Fig. 2B and C displayed, the WebLogo diagram of the selected PQSs showed that extensive guanine residues involved in PQSs were highly conserved. Interestingly, PQS-VP2 was found to be highly conserved among EV-A71 strains genomes, however, there was little guanine conserved among all species. Thus, PQS-VP2 was a PQS exclusive to EV-A71 strains genomes. As a consequence, PQSs might be key elements in the viral cycle and be selected for during viral genome evolution.

### Biophysical characteristic patterns of EV-A71 PQSs.

**FIG 3:**
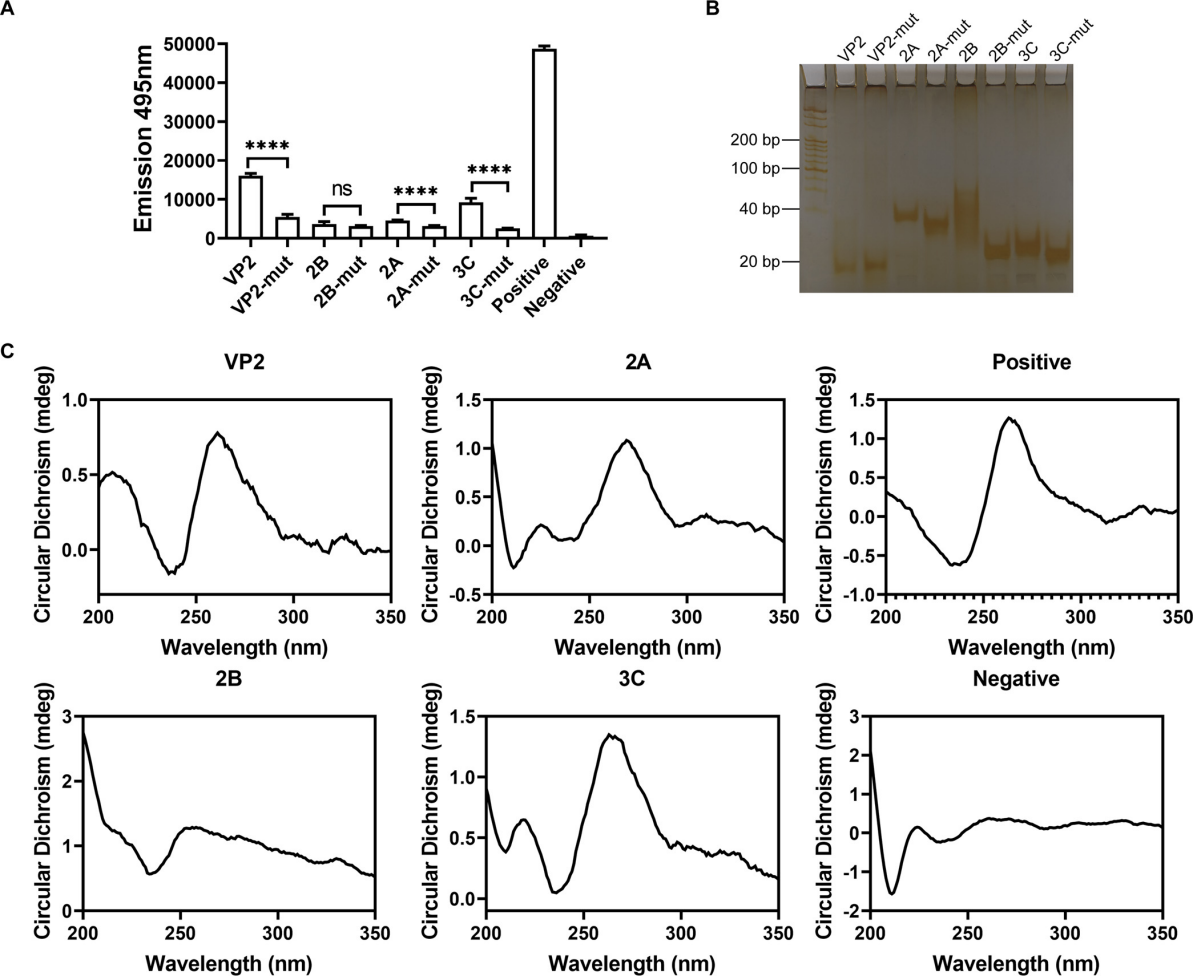
Characterization of the G-quadruplex sequences. (A) ThT fluorescent assay of the selected G-quadruplex and G-mut sequences analyzing the extent of formation. The emission and excitation were at 495 nm and 425 nm, respectively. ****P < 0.0001 and ns, no significance based on the Student's *t* test. Error bars represent the SD. (B) Native gel electrophoretic analysis of PQSs and G-mut formation. 0.32 nmol RNA solution was prepared in 10 mM Tris HCl (pH = 7.4) buffer with 100 mM KCl. (C) CD spectral analysis for G-quadruplexes oligonucleotide showing RNA topology generated by GraphPad Prism software version 8.0. The wavelength range was from 200 to 350 nm. RNA oligonucleotides were diluted to 20 μM final concentration in 10 mM Tris HCl (pH = 7.4) buffer with 100 mM KCl.

We initially assessed the actual ability of selected PQSs to form G-quadruplex structure *in vitro* through Thioflavin T (ThT) fluorescent assay (30). The fluorescence emission at 495 nm was applied after excitation at 425 nm. The four PQSs could indeed form G-quadruplex structures as shown in Fig. 3A, the degree of G-quadruplex structure formation varied in different regions. The fluorescence emission in G-mut sequences was lower than that in G-quadruplex sequences except for PQS-2B. Native polyacrylamide gel electrophoresis (PAGE) measurements were carried out for further verifying PQSs formation. Compared to PQSs, single-stranded nucleic acids corresponding to PQS-mut migrated at a different rate (Fig. 3B). Combined with result of THT assay, PQS-2B may not form a stable G-quadruplex structure. To further support the existence of G-quadruplex structure in the EV-A71 genome, we conducted a circular dichroism (CD) spectrum experiment. CD spectra of PQS-VP2, 2A, 3C exhibited a positive peak at around 260 nm and a negative peak at near 240 nm of a typical parallel G-quadruplex structure (Fig. 3C). Consistent with THT assay result, PQS-2B displayed a broad plateau in the 260–280 nm region, which indicated a weak G-quadruplex formation (Fig. 3C).

### Binding of G-quadruplex ligands to EV-A71 PQSs stabilized G-quadruplex structures.

**FIG 4:**
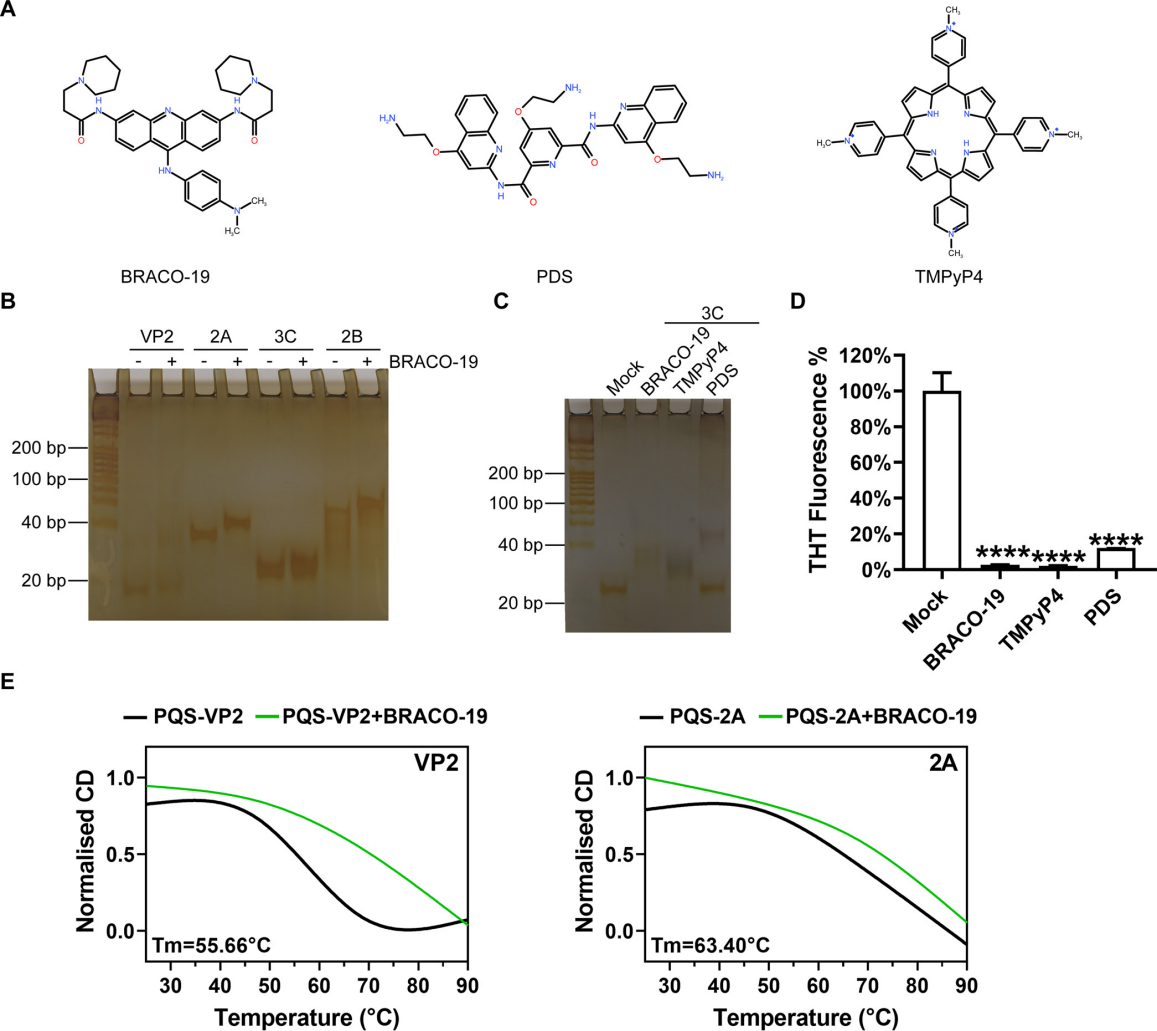
Binding and stabilization of G-quadruplex structure by G4 ligands. (A) The chemical structures of BRACO-19, PDS, and TMPyP4 generated by KingDraw v2.0.2. (B) Native gel electrophoresis of G-quadruplex structures. The annealed 0.4 nmol G-quadruplex RNA solution with BRACO-19 is at ratios of 1:1 M. (C) Native gel electrophoresis of G-quadruplex structures in the presence of G4 ligands. The 30 μM ligands were added to the annealed 0.24 nmol G-quadruplex RNA solution. (D) G-quadruplex RNA oligonucleotides pre-incubated with three compounds, the fluorescence was measured after the addition of ThT and normalized to the initial fluorescence signal. ****P < 0.0001 based on the Student's *t* test. Error bars represent the SD. (D) Comparison of CD melting profiles at 265 nm wavelength for G-quadruplex structures in the presence (green) of BRACO-19 generated by GraphPad Prism software version 8.0. Tm values of each G-quadruplex in the absence of ligand are represented in the left bottom corner.

The G-quadruplex structures can be stabilized by G-quadruplex ligands (31), such as BRACO-19, pyridostatin (PDS), 5,10,15,20-tetrakis-(N-methyl-4-pyridyl)porphyrin (TMPyP4) (Fig. 4A), which inhibited viral replication at the step of transcription, translation, or genome replication. We next examined whether BRACO-19, PDS, and TMPyP4 could affect the behavior of EV-A71 G-quadruplex structures. The relative affinity and selectivity of ligands binding to PQSs were confirmed in native PAGE. We found BRACO-19 treatment slowed down the movement of PQSs in electrophoresis (Fig. 4B). When we chose a represent PQS under the same concentration treatment of three compounds, the degrees of the slowed movement was distinct, indicating the different binding affinity of G-quadruplex ligands to PQSs (Fig. 4C). These results were also confirmed by THT assay. THT fluorescent signal had an evident decrease under low concentrations treatment. However, three compounds decreased the fluorescence to disparate extents at the same concentration (Fig. 4D).

In addition, we performed thermal denaturation experiments to illustrate the stability of G-quadruplex structure following the addition of the BRACO-19. When BRACO-19 was added in double the molar concentration to the annealed G-quadruplex RNA solution, the stabilization of the G-quadruplex structures made it difficult to unfold, leading to the higher melting temperature (*T_m_*) (Fig. 4E). This result further demonstrated the higher stability of the nucleic acid conformation and the strong binding of ligand and EV-A71 G-quadruplexes.

**FIG 5:**
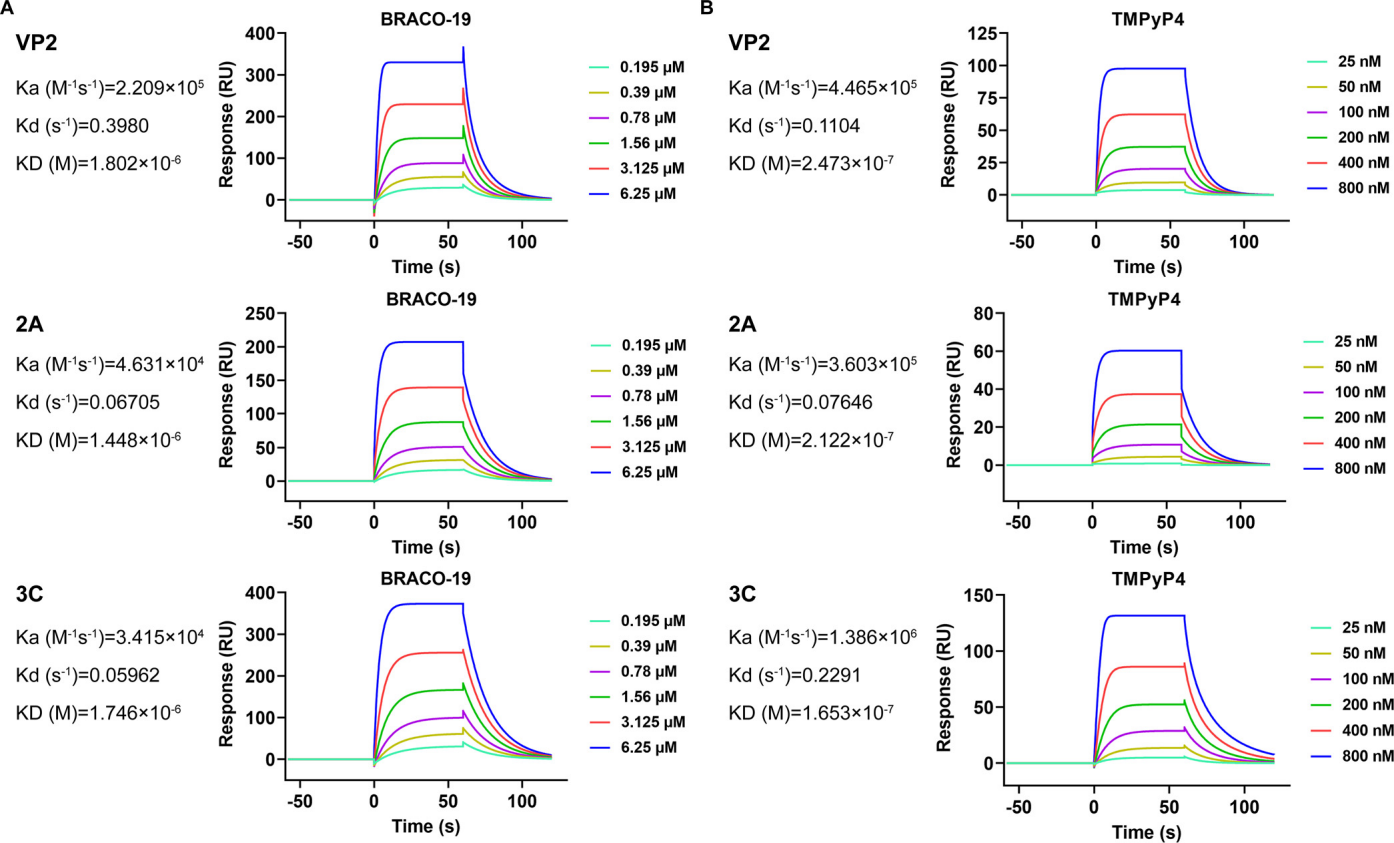
Analysis of G-quadruplex-ligands interactions by SPR spectroscopy. The SPR analysis of G-quadruplex structure showed their binding affinities toward BRACO-19 (A) and TMPyP4 (B) generated by GraphPad Prism software version 8.0.

Next, the binding affinities of G-quadruplex ligands, BRACO-19 and TMPyP4, toward represent PQSs forming G-quadruplex structure were developed by a surface plasmon resonance (SPR) biosensor. Compound TMPyP4 exhibited 7.3, 6.8, and 10.6–fold higher binding affinity than BRACO-19 toward VP2, 2A, and 3C, respectively (Fig. 5A and B). The higher association constants suggested that TMPyP4 was a better ligand toward EV-A71 G-quadruplexes.

### Restriction of gene transcription through G-quadruplex structure stabilization.

**FIG 6:**
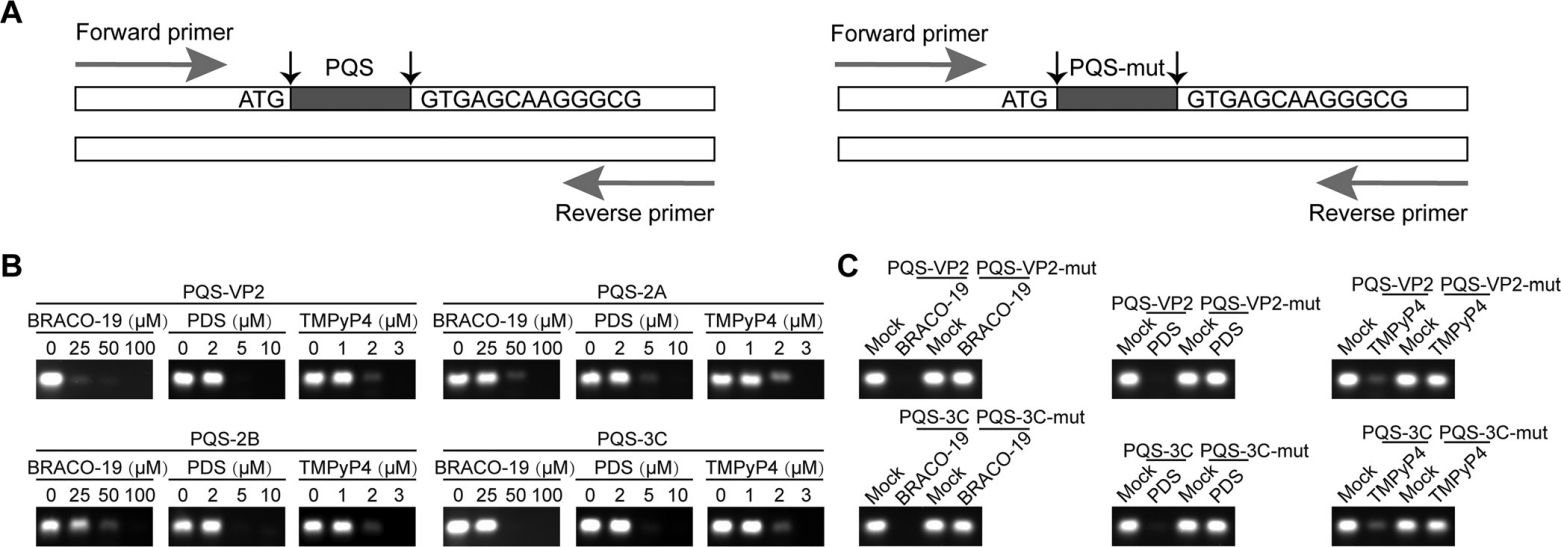
Effection of G‐quadruplex structure on gene transcription. (A) Schematic diagram showing plasmids construction and primer extension assay. The pEGFP-C1 vector was engineered with G-quadruplex or PQS‐mut sequences sited after the ATG start codon. (B) Primer extension assay showed that the intensity of PCR products was decreased with the increasing concentration of BRACO‐19, PDS, and TMPyP4. (C) Primer extension assay showed that the intensity of PCR products from PQS-mut remained the same in the presence of BRACO‐19, PDS, and TMPyP4.

Generally, the secondary structure had a significant impact on protein expression and function (32, 33). G-quadruplex structures were likely involved in modulating biological pathways, for instance, genome instability, transcription, translation and replication (34). We performed primer extension assays to investigate whether stabilization of G-quadruplex structure in EV-A71 genome affected gene expression. PQS or PQS-mut was inserted after the ATG start codon of the pEGFP‐C1 vector (Fig. 6A). When G-quadruplex structure was stabilized by PQS binders, the gene transcription was terminated to some extent and PCR products was declined in a dose-dependent manner (Fig. 6B). However, the stabilizer had no effect on PQS-mut sequences (Fig. 6C). These data demonstrated that PQS ligands hampered gene transcription.

### Inhibition of viral replication by G-quadruplex ligands.

**FIG 7:**
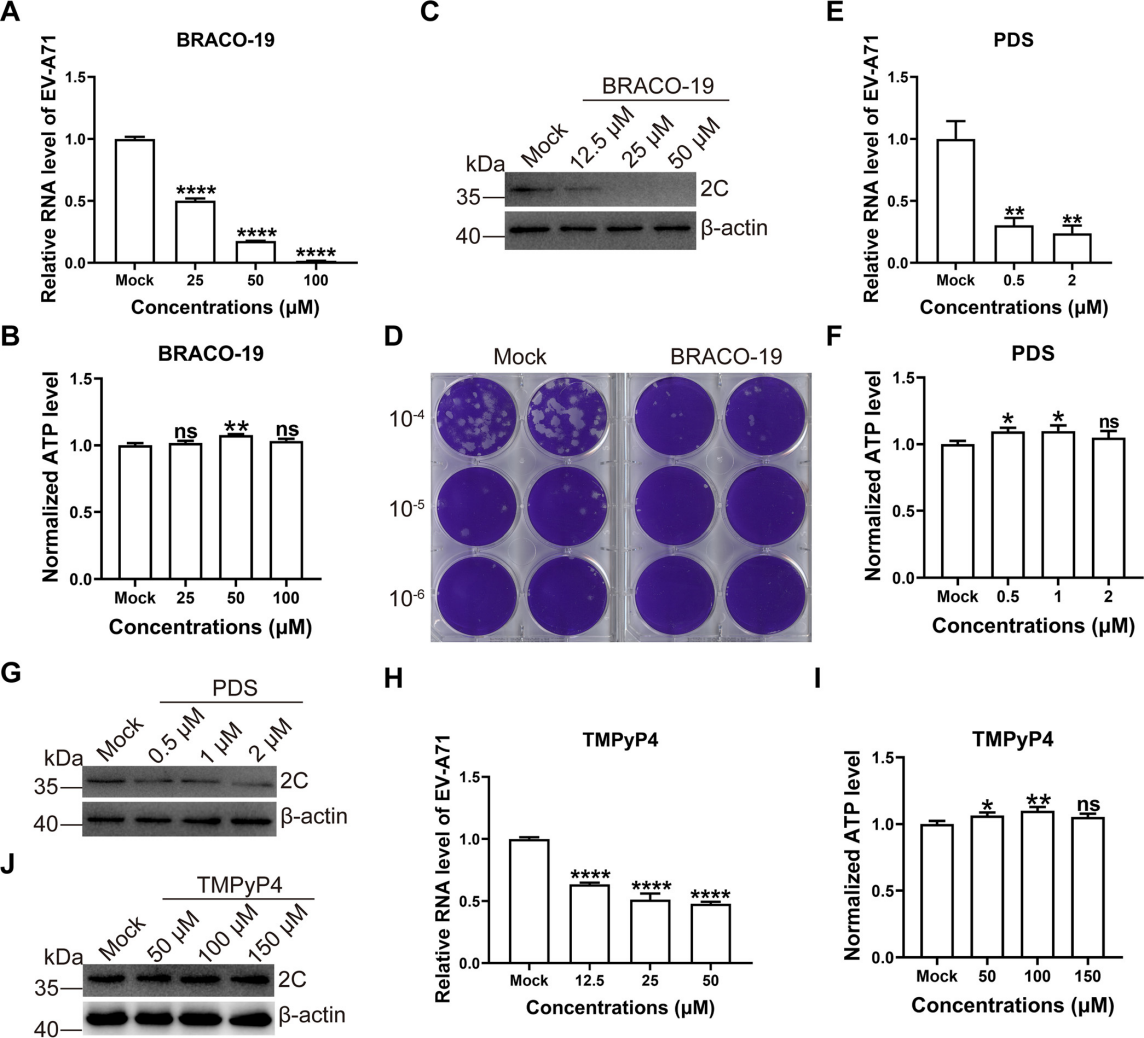
Inhibition of EV-A71 replication by G-quadruplex ligands. (A) RD cells were infected with EV-A71 strain FY23 (MOI = 3) for 1h and then treated with G-quadruplex ligands BRACO-19 for 5h. The viral RNA was detected with quantitative PCR. Data were presented with average ± SD based on the Student's *t* test. ****, *P* < 0.0001. (B) Effects of BRACO-19 on cell viability of RD cells (A) were determined by measuring ATP content using a CellTiter-Glo assay. Data were presented with average ± SD based on the Student's *t* test. ns, no significance; **, *P* < 0.01. (C) RD cells were infected with EV-A71 strain FY23 (MOI = 3) for 1h then treated with BRACO-19 overnight. The lysates were immunoblotted for 2C and β-actin. (D) RD cells infected with EV-A71 strain FY23 (MOI = 3) for 24h in the presence of 50 μM BRACO-19 and extracellular virus titer was measured by plaque assay in RD cells. (E) RD cells were infected with EV-A71 strain FY23 (MOI = 3) for 1h and then treated with G-quadruplex ligands PDS for 5h. The viral RNA was detected with quantitative PCR. Data were presented with average 6 SD based on the Student's *t* test. **, *P* < 0.01. (F) ATP levels in cells (E) were measured by CellTiter-Glo luminescent cell viability assay. Data were presented with average ± SD based on the Student's *t* test. ns, no significance; *, *P* < 0.05. (G) RD cells were infected with EV-A71 strain FY23 (MOI = 3) for 1h then treated with PDS overnight. The lysates were immunoblotted for 2C and β-actin. (H) RD cells were infected with EV-A71 strain FY23 (MOI = 3) for 1h and then treated with G-quadruplex ligands TMPyP4 for 5h. The viral RNA was detected with quantitative PCR. Data were presented with average ± SD based on the Student's *t* test. ****, *P* < 0.0001. (I) Effects of TMPyP4 on cell viability of RD cells (H) were determined by measuring ATP content using a CellTiter-Glo assay. Data were presented with average ± SD based on the Student's *t* test. ns, no significance; *, *P* < 0.05; **, *P* < 0.01. (J) RD cells were infected with EV-A71 strain FY23 (MOI = 3) for 1h then treated with TMPyP4 overnight. The lysates were immunoblotted for 2C and β-actin.

We next explored whether stabilization of G-quadruplex structures in the EV-A71 genome upon G-quadruplex ligands may modulate replication of viral genomes. Human rhabdomyosarcoma (RD) cells were infected with EV-A71 strain FY23 at a multiplicity of infection (MOI) of 3 for 1h and then treated with varied concentrations of the drugs. The level of viral RNA was assessed by using quantitative real-time PCR. BRACO-19 treatment significantly reduced EV-A71 replication as evidenced by the decrease in virus RNA level without impact on cell viability (Fig. 7A and B). Then, we examined the EV-A71 2C protein expression levels in virus-infected cells in the presence of BRACO-19. RD cells were treated with varied concentrations 20h postinfection. The 2C protein was shown a marked reduction in expression with increasing concentrations of BRACO-19 (Fig. 7C). The role of BRACO-19 (50 μM) on EV-A71 replication was further confirmed with plaque assay, which implicated more than an order of magnitude decrease in infectious virus titer in comparison with the mock-treated cells (Fig. 7D). Treatment with PDS restricted EV-A71 replication supported by quantitative PCR while the cell viability was not changed (Fig. 7E and F). However, PDS only slightly reduced viral 2C protein expression (Fig. 7G). PDS exhibited an evident effect under higher concentration probably, whereas higher concentration displayed cytotoxic activities to cells. Treatment with TMPyP4 also reduced EV-A71 replication supported by quantitative PCR while the cell viability was not changed (Fig. 7H and I). Although TMPyP4 at the maximum concentration of 150 μM showed no effect on cell viability, the viral 2C protein had no significant change (Fig. 7J). TMPyP4 was shown to be less stable than BRACO-19 (35). We speculated that overnight incubation at 37°C might cause the lost of activities of TMPyP4. Although TMPyP4 had a higher binding affinity to PQSs as shown in Fig. 4, BRACO-19 might be more suitable for developing novel antiviral drugs according to the stabilities of compounds.

## DISCUSSION

The research on G4s stems from its unique biological structure and its wide distribution in organisms. At present, it has been considered a basic structural feature of the genome. The G4s structure *in vivo* has a certain dynamics and functions in many important cellular biological processes. It is also involved in maintaining the stability of the genome. In recent years, G-quadruplex structures have been considered a new antiviral strategy targeting viral virulence (36). Our study provides a comprehensive overview of G-quadruplex structures in HEVs genomes. Through the computational prediction, we identified a series of G-quadruplex sequences in EV-A71 genome. We selected four sequences to analyze G-quadruplex formation. Multiple methods demonstrated that BRACO-19, PDS and TMPyP4 could bind and stabilize the G-quadruplexes. We also demonstrated that BRACO-19 could inhibit EV-A71 replication in a dose-dependent manner.

N, N’-(9-((4-[dimethylamino]phenyl)amino)acridine-3,6-diyl)bis (3-[pyrrolidin-1-yl]propan-amide), named as BRACO-19, is a trisubstituted acridine molecule employed as one of the most characterized PQS ligands. Since the nitrogen atoms in the heterocyclic scaffold could be protonated under physiological conditions, the electronic defects in the chromophore exhibited an increase, resulting in enhancement of the PQS interaction (37). Moreover, the binding to G-quartets resided on a central planar pharmacophore through π-π interactions and the two side chains with a tertiary amine interacted with the grooves (38). The structure provided the interplay basic of BRACO-19 and PQS. Compound TMPyP4 was another extensively studied G4-binding molecules. Interestingly, TMPyP4 exerted opposite effect on PQS structures, stabilization, and destabilization. TMPyP4 increased G4 stability through stacking its aromatic core on the upper or lower G4 tetrad (38), whereas TMPyP4 induced G4 unfolding through two conformation states composed of groove-bound and top-face-bound (39). The nature of binding mode of TMPyP4 to G4 remains controversial. It may also be one of reasons why TMPyP4 had not significantly effect on inhibiting viral protein. PDS comprised an electron rich aromatic surface, the potential for a flat conformation, an ability to participate in hydrogen bonding, and the rotatable bonds, which made it feasible to interact with PQS (40). However, PDS showed cytotoxic activities under higher concentration.

G-quadruplex structures were still a considerable target for design and development of small-molecule ligands. The common structural characters of these ligands had a heterocyclic core that bind with G-quadruplex structures. The best representative compound of bisquinolinium, PhenDC3, prevented viral infection of KSHV and EBV (41, 42). Some ligands belonging to the class of perylenes and naphthalene diimides, PIPER or c-exNDIs, were tested against PQS of HIV-1 and HSV-1 (43–46). In the future, a diverse set of PQS ligands mentioned here could be analyzed for inhibiting EV-A71 replication to develop therapeutics.

G4s exerts its biological functions by affecting biological processes such as DNA replication, transcription and translation. On the other hand, there are also a series of proteins to relieve the effect of G4s. Some of these proteins are closely related to the stability of telomeres, and some are related to the expression regulation of proto-oncogenes. The identification of G4 binding proteins is important to further clarify the biological function of G4. At present, several host proteins have been identified to associate with viral G4, which are involved in the formation or function of G4 structure. Nucleolin could associate with G4 encodes by HCV and EBV to regulate HCV replication (19) and immune evasion (41). CNBP promoted the unfolding of G-quadruplex in SARS-CoV-2 RNA genome (47). In the future, it is necessary to identify host proteins associated with EV-A71 G4 and to further study the practical significance and biological function of the interaction between host proteins and viral G4.

The biological function of G4 is fulfilled through dynamic folding and opening. In cells, G4 can fold spontaneously, but its opening is completed by special helicases (48). NS3 protein of *Flaviviridae* family of viruses containing an unique helicase domain could unwind G-quadruplex folding (23). It will be interesting to investigate whether EV-A71 2C helicase could interact with or unwind G-quadruplex sequences from EV-A71. Helicase inhibitors cannot only inhibit RNA unwinding to inhibit virus replication, but also exert inhibitory effect through the unfolding of G-quadruplex. Therefore, helicase inhibitors may also act as potential drug targets through G-quadruplex structure. We have reason to infer that the discovery of G4 ligands with clinical significance and FDA approved helicase inhibitors will more effectively find clinical drugs for the treatment of human enteroviruses.

Viruses rely on host cells to complete their life cycle and affect all organisms on the earth. It is one of the main causes of human diseases and has a serious impact on human health and social economy. Therefore, the development of virus detection tools is of great significance to prevent virus infection and diseases caused by viruses. Virus genome plays an important role in the life cycle of virus and provides an effective target for virus detection. As a stable nucleic acid secondary structure formed by the folding of guanine rich nucleic acid, G4 participates in the regulation of a variety of biological functions and plays a key role in important cell physiological processes. The rapid development of G4 specific probes provides an indispensable key element for the construction of new biosensor methods, the detection of G4 structure and cell imaging. At present, G4/probe complex as a signal output unit has been widely used in biosensor. Some methods can realize label free detection of a variety of virus gene sequences and show great potential in the field of diagnosis and clinical analysis of virus-related diseases. In addition, G4 widely exists in the virus genome and becomes a potential imaging target in the virus gene, which provides a new idea for virus visualization. Research progresses of biosensor methods based on G4 probe in virus genome detection have been made recently. HCV RNA could be detected by ThT-NE derived in living cells (49). Infrared G-quadruplex mimics of fluorescent proteins (igMFP) targeting HCV G4 were generated and could visualize HCV in living cells and living mice (50). In the future, the development of EV-A71 G4 specific fluorescent molecular probes is expected to provide support for the visual imaging analysis of EV-A71 genome in living cells.

## MATERIALS AND METHODS

### G-quadruplex sequence mapping.

The available genomes of the Enterovirus family and EV-A71 strains were retrieved from the NCBI Genome database. The complete sequences from Enterovirus A(6), Enterovirus B(6), Enterovirus C(2), Enterovirus D(3), and Rhinovirus A(3), Rhinovirus B(2), and Rhinovirus C(2) were analyzed. The corresponding GenBank accession numbers are listed in Table 1. QGRS mapper was used to predict the PQSs from EV-A71 strain FY23 with four repeats of G groups and a minimum G group of 2 (29). The maximum length was up to 30 nucleotides with a loop size from 0 to 12 nucleotides. Conservation analysis was performed on selected Enterovirus family and EV-A71 strains genomes in database using the MEGA X software (51). LOGO representation of base conservation was obtained by the WebLogo software online (52).

**TABLE 1:** GenBank accession numbers of Enterovirus family and EV-A71 strains

Enterovirus	GenBank accession no.
Enterovirus A	JX867330.1, KP289435.1, MH118086.1, EU812515.1, MH118030.1, KT277550.1
Enterovirus B	LN854562.1, EF174468.1, FJ357838.1, AY302560.1, KF874626.1, DQ902713.1
Enterovirus C	JX174177.1, KX162693.1
Enterovirus D	MN240507.1, DQ201177.1, MT081371.1
Rhinovirus A	DQ473498.1, FJ445175.1, FJ445111.1
Rhinovirus B	DQ473490.1, FJ445112.1
Rhinovirus C	OK017929.1, MN369038.1
EV-A71 strains	DQ341355.1, DQ341356.1, DQ341357.1, DQ341358.1, DQ3413559.1, DQ341361.1, EU812515.1, GQ994989.1, GU459070.1, AB550332.1, HM245927.1, AB550333.1, AB550340.1, AB550341.1, JN992283.1, JN992285.1, KC436270.1, AB747375.1, MG214681.1, LR027524.1, LR027531.1, LC627083.1, MT708803.1, MT708802.1, MT708801.1, MT708800.1, MT708799.1, MT081373.1, LR027546.1, LR027542.1, MF662685.1, AB550335.1, DQ452074.1, AB550334.1, MT188611.1, MN966512.1, KF154355.1, HQ188292.1, DQ341368.1, DQ341367.1, DQ341366.1, DQ341365.1, AF352027.1, AF316321.2, MT241233.1, MT360998.1, MT360997.1, MT360996.1, MT360995.1, MT360994.1, MT360993.1, MT360992.1, MT360991.1, MT360990.1, MT360989.1, MT360988.1, MT360987.1, MT360986.1, MT360985.1, MT360984.1,

### ThT fluorescent assay.

BRACO-19, PDS and TMPyP4 powders were purchased from Sigma, MedChemExpress and TCI, respectively. The compounds were diluted in 10 mM Tris HCl (pH = 7.4) and 100 mM KCl buffer. The sequences of G-quadruplex and G-mut were listed in Table 2. Synthetic RNA oligonucleotides in powder were diluted to 2 μM final concentration in 10 mM Tris HCl (pH = 7.4) and 100 mM KCl buffer, heated at 95°C for 5 min and slowly cooling down to room temperature. The RNA solution was incubated at 4°C overnight in the absence or presence of compounds. ThT powder was bought from Aladdin Industrial Corporation. Oligonucleotides and ThT were mixed at 1:0.5 M ratio to a final concentration of 1 and 0.5 μM, respectively. Fluorescence emission was recorded at 495 nm after excitation at 425 nm using a TECAN SPARK microplate reader (30).

**TABLE 2:** Sequences for THT fluorescence and CD spectrum

Name	G-quadruplex or G-mut sequence
VP2	5′-UGGACAGUGGCAGGCGGUACAGGGA-3′
VP2-mut	5′-UGGACAGUAGCAGGCAGUACAGGGA-3′
2A	5′-CGGUGGCAAUGGGCUCGUUGGA-3′
2A-mut	5′-CGGUAGCAAUGAGCUCGUUGGA-3′
2B	5′-CGGUUGUCAUGGCAGUCCUUGGGCUUGGA-3′
2B-mut	5′-CGGUUGUCAUAGCAGUCCUUAGGCUUGGA-3′
3C	5′-UGGUUGUCGGUAUUCACAUUGGUGGCAAUGGA-3′
3C-mut	5′-UGGUUGUCAGUAUUCACAUUAGUGGCAAUGGA-3′
Positive	5′-AGGGCGGUGUGGGAAGAGGGAAGAGGGGGAGGCAG-3′
Negative	5′-GCGCGCGCUUUUGCGCGCGC-3

### Native PAGE.

The compounds were diluted in 10 mM Tris HCl (pH = 7.4) buffer with 100 mM KCl. The annealed G-quadruplex RNA solution was placed at 4°C overnight in the absence or presence of compounds. Native PAGE experiment was carried out on acrylamide gel (15%) and run at 1 × TBE buffer containing 10 mM KCl for 2 h and was silver stained.

### CD.

Synthetic G-quadruplex RNA oligonucleotides were diluted to 20 μM final concentration in 10 mM Tris HCl (pH = 7.4) and 100 mM KCl buffer. CD measurements were carried out with a Jasco J-1500 Spectropolarimeter with a Peltier Temperature Controller using a quartz cuvette with 0.1 mm optical path length and a sample volume of 40 μL. CD spectrum was performed from a wavelength range of 200 to 400 nm. CD melting spectroscopy was collected at 265 nm and 290 nm at a temperature range of 25°C to 90°C with the scanning speed (20 nm/min) and the intervals temperature (1°C). The ligand was added gradually in double the molar concentration of G-quadruplex RNA solution. The data were obtained and normalized using origin2021 software.

### SPR.

Biotinylated RNA samples were annealed and folded in 10 mM Tris HCl (pH = 7.4) and 100 mM KCl buffer overnight. SPR experiments were performed on the Biacore T200 platform. All experiments were performed at 25°C. Biotinylated G-quadruplexeswere immobilized on Sensor chip SA. Binding analysis was conducted at a flow rate of 30 μL/minin the running buffer (10 mM Tris HCl pH = 7.4 and 100 mM KCl). Compound solutions were prepared within the running buffer by serial dilutions from stock solutions. The final graphs were obtained by subtracting blank sensor grams from quadruplex sensor grams. Association and dissociation rates and constants of the G-quadruplex-ligand complexes were determined using Biacore evaluation software. The data were globally fitted to a 1:1 binding model.

### Primer extension assays.

We constructed PQS-VP2, PQS-2A, PQS-2B, PQS-3C, PQS-VP2-mut, and PQS-3C-mut plasmids. G-quadruplex sequences and their mutants were cloned after the ATG start codon of the pEGFP‐C1 vector. These plasmids were used as polymerase chain reaction (PCR) templates. The forward and reverse primer for PCR assays were 5′-CGCTAACGCTAACGGTCGTCAAC-3′ and 5′-GGCAACACCCCGGTGAACATCT-3′. Primer extension assays were performed at 20 μL final volume of PCR mixture, including 2×EasyTaq PCR SuperMix (AS111 TransGen Biotech), 0.4 μL each oligonucleotide, 0.4 μL forward and reverse primer, and varied concentration of BRACO-19, PDS, or TMPyP4. The reaction products were subjected to 2% agarose gel electrophoresis run in 1× Tris-Acetate-EDTA buffer. The gel was visualized by GelStain (GS101 TransGen Biotech) staining and captured using the BIO-RAD GelDoc Go.

### Quantitative real-time PCR.

RD cells were seeded at a density of 3 × 10^5^ cells per well in 24-well cell culture plates 1 day before the experiment. Cells were infected with the virus at an MOI of 3 for 1h at 37°C. Then, the inoculum was removed and replaced with nonfetal bovine serum medium containing various concentrations of BRACO-19, PDS, or TMPyP4. After incubation for 5h, total RNA from infected cells was isolated using TRIzol RNA isolation reagent (Thermo Fisher Scientific). After RNA extraction, according to the manufacturer’s instructions, qRT-PCR was performed using a TransStart Green qPCR SuperMix UDG kit (Transgen, China) with the LightCycler 480 RealTime PCR System (Roche, Basel, Switzerland). The forward and reverse primers for EV-A71 were 5′-GCAGCCCAAAAGAACTTCAC-3′ and 5′-ATTTCAGCAGCTTGGAGTGC-3′. The forward and reverse primers for GAPDH were 5′-GGAGCGAGATCCCTCCAAAAT-3′ and 5′-GCTGTTGTCATACTTCTCATGG-3′.

### Western blotting.

RD cells were seeded at a density of 3 × 10^5^ cells per well in 24-well cell culture plates 1 day before the experiment. Cells were infected with the virus at MOI of 3 for 1h at 37°C. Then, the inoculum was removed and replaced with nonfetal bovine serum medium containing various concentrations of BRACO-19, PDS, or TMPyP4. After incubation overnight, the cell pellet was resuspended in lysis buffer and incubated on ice for 10 min. The lysates were clarified by centrifugation at 12000 rpm for 15 min at 4°C. 20 μL cell lysates were separated by electrophoresis on a required percentage of PAGE containing sodium dodecyl sulfate (SDS). Primary antibodies included rabbit EV-A71 2C antibody generated by our lab (53) and mouse β-actin antibody (catalogno. A2228; Sigma-Aldrich, St. Louis, MO).

### Cell viability assays.

Cytotoxicity was assessed using a CellTiter-Glo Luminescent cell viability assay kit (Promega, Madison, WI) according to the manufacturer’s instructions. In brief, the cells were lysed by adding an amount of CellTiter-Glo Reagent at room temperature. After 5 min shaking and 10 min incubation, luminescence was measured using a TECAN SPARK microplate reader.

### Plaque assay.

EV-A71 strain FY23 were propagated in DMEM supplemented with 2% FBS in RD cells. All viruses were titrated by plaque assay. RD cells were seeded into a 24-well cell culture plate at a density of 3 × 10^5^ cells per well. Cells were infected with the virus at an MOI of 3 for 1 h at 37°C. Then, the inoculum was removed and replaced with a non-FBS medium containing 50 μM BRACO-19. Afterwards, the infected cells were further incubated until 24h and the supernatants were collected and determined for virus titration. RD cells in 6-well plate were grown to 90–100% confluence and the harvested supernatant samples were serially diluted and inoculated to the cells for 2 h at 37°C. After virus adsorption, the cells were washed with PBS twice and covered with 1.4% agarose/PBS mixed with 2× DMEM/0.4% FBS at 1:1 ratio. After incubation at 37°C for 96 h, the plates were fixed with 4% paraformaldehyde for 1 h. The agar was removed and cells were stained with 0.1% crystal violet for 10 min. And then the plaque was visualized.

### Statistical analysis.

The statistical data were analyzed using GraphPad Prism software version 8.0. The two-tailed Student's *t* test was used to determine significant differences between treated and control groups. All values are depicted as mean ± standard deviation (SD). *P* value of no less than 0.05 was considered statistically not significant (ns). *, *P* < 0.05; **, *P* < 0.01; ***, *P* < 0.001; ****, *P* < 0.0001.

### Data availability.

All data generated or analyzed during this study are included in this article. Constructs Data are available via requests to the corresponding author.
